# The Role of Greenness in Heat-Related Mortality: A Systematic Review and Meta-Analysis

**DOI:** 10.5334/aogh.5216

**Published:** 2026-09-25

**Authors:** Ana Perez-Moreno, Maria Martinez-Andres, Blanca Notario-Pacheco, Irene Marcilla-Toribio, Shkelzen Cekrezi, Lidia Lucas-de la Cruz, Antonio Miguel Caraballo-Betancort, Bruno Bizzozero-Peroni

**Affiliations:** 1 Health and Social Research Center, Universidad de Castilla-La Mancha-Campus de Cuenca, 16071, Cuenca, Spain; 2 Research Group Health, Gender, and Social Determinants, Universidad de Castilla-La Mancha-Campus de Cuenca, 16071, Cuenca, Spain; 3 Research Group Health, Gender, and Social Determinants, Instituto de Investigación Sanitaria de Castilla-La Mancha (IDISCAM), Spain; 4 Faculty of Nursing of Albacete, Universidad de Castilla-La Mancha-Campus de Albacete, 02071, Albacete, Spain; 5 Faculty of Nursing of Cuenca, Universidad de Castilla-La Mancha-Campus de Cuenca, 16071, Cuenca, Spain; 6 Higher Institute of Physical Education, Universidad de la República, 20000, Maldonado, Uruguay; 7 Department of Neurobiology, Care Sciences and Society, Aging Research Center, Karolinska Institutet and Stockholm University, 17165, Stockholm, Sweden

**Keywords:** green spaces, environment, extreme heat, heat wave, differential mortality

## Abstract

*Background:* Rising temperatures and the frequency of heat waves are challenges that have an impact on the health and mortality of populations. A high level of greenness is emerging as a strategy to mitigate the effects of extreme heat.

*Objective*: To assess whether higher levels of greenness are associated with reduced mortality compared with lower greenness during periods of extreme heat.

*Methods:* In this systematic review and meta-analysis, we searched MEDLINE, SCOPUS, WEB OF SCIENCE, CINHAL, Environment Complete, and GreenFILE. Observational studies were included. Pooled risk ratios (RRs) with 95% confidence intervals (CIs) were calculated via a random-effects model.

*Results:* The systematic review and meta-analysis included 11 articles. These cover data from China, France, Germany, Jordan, Switzerland, and the USA, as well as a global analysis covering 24 countries. The meta-analysis suggested that heat waves significantly increased mortality in areas with the lowest greenness (RR = 1.33; 95% CI: 1.15–1.55; *I*^2^ = 99.4%) and in areas with the highest greenness (RR = 1.18; 95% CI: 1.03–1.36; *I*^2^ = 97.9%). The pooled ratio of RR (RoR) indicated that the association was significantly stronger in low-greenness areas than in high-greenness areas (RoR = 1.11; 95% CI: 1.06–1.15; *I*^2^ = 90.5%), indicating that the heat–mortality association was 11% stronger in low-greenness areas. The pooled associations varied descriptively across subgroup analyses, although no statistically significant differences were observed between geographical regions or study settings.

*Conclusion:* Higher temperatures were associated with increased mortality in both low- and high-greenness areas; however, the heat–mortality association was 11% stronger in low-greenness areas. These findings suggest that greater greenness may attenuate vulnerability to extreme heat, although the limited evidence base and considerable between-study heterogeneity warrant cautious interpretation.

## Introduction

Climate change and global warming are two of the most pressing challenges facing our society today [1, 2]. These factors impact global public health by increasing the number of extreme weather events [3]. One of the most common of these is heat waves and extremely high temperatures [4, 5]. The probability of the near-surface temperature exceeding 1.5°C above preindustrial levels for a minimum of one year between 2023 and 2027 is 66% [6]. Rising temperatures pose a growing threat to public health, increasing mortality and morbidity rates [7, 8]. There has been an increase in heat-related deaths in recent decades [9]. Projections indicate that, in the scenario of a 3°C temperature increase, the number of temperature-related fatalities could reach 54,974 by the year 2100 [10].

The impact of climate change on mortality and morbidity during heat waves has been studied in relation to health and temperature. The human body maintains thermal homeostasis by adapting to changes in ambient temperature through physiological mechanisms involving the circulatory, respiratory, and nervous systems [11]. Nevertheless, extreme temperatures can have a significant impact on the human body, which may be unable to adapt properly [12]. As a result, the body’s inability to regulate temperature effectively increases the risk of heat-related illnesses and mortality [13–15]. In addition to the direct effects of extreme heat exposure, elevated temperatures are also associated with indirect risks, including an increased incidence of accidents [11].

Several sociodemographic, health, physical, socioeconomic, and institutional factors influence vulnerability to heat-related health outcomes. Older adults are among the most vulnerable populations due to age-related impaired thermoregulation [12]. For example, densely populated urban areas are particularly susceptible to the urban heat island effect, in which air temperatures are significantly higher than those in surrounding rural regions [16, 17].

In the context of a warming planet, where extreme heat is becoming more frequent and intense, the vulnerability of populations to extreme heat has become a pressing concern on policy and research agendas. In response to this challenge, nature-based solutions have been identified as a key strategy for tackling climate change. Recent research has demonstrated that green spaces and vegetation play a crucial role in mitigating the health effects of extreme heat [18]. In addition, a series of studies indicates that green spaces, including parks, gardens, and green rooftops, contribute to a reduction in mortality attributable to extreme heat [19–21]. For example, research conducted in European cities shows that an increased presence of green spaces can reduce the impact of extreme heat on vulnerable groups, such as the elderly and those with preexisting health conditions [22].

Previous systematic reviews and meta-analyses have reported a protective association between higher levels of greenness and a lower risk of all-cause mortality [18, 23, 24]. These findings imply that green spaces may help to mitigate the adverse health effects of rising temperatures. However, a systematic review and meta-analysis focusing specifically on the relationship between greenness and heat-related mortality are needed to provide a more comprehensive and robust understanding. This study aims to synthesize the available evidence from observational studies to offer a clearer perspective on the potential role of greenness in reducing mortality during extreme heat events.

## Material and Methods

### Search strategy

This systematic review and meta-analysis was conducted according to the 2020 Preferred Reporting Items for Systematic Reviews and Meta-Analyses statement (Table S1. PRISMA Checklist) [25] and followed the recommendations of the Cochrane Collaboration Handbook [26]. The protocol was registered in PROSPERO (CRD42024559902).

A systematic search for studies was conducted through five electronic databases, including MEDLINE, SCOPUS, WEB OF SCIENCE, CINHAL “Environment complete,” and “GreenFILE.” An initial systematic search was conducted in January 2024 and was later updated to include studies published through November 26, 2025. The search for information was guided by the following research question: Is greater greenness associated with lower heat-related mortality? To address this question, the following search strategy was applied: (heat OR “heatwave” OR hot OR “high temperature”) AND (greenness OR greenery OR “residential greenness” OR “NDVI” OR “urban green space” OR “green space”) AND (mortality OR “heat-mortality”). No other filters were applied. During the identification, selection, and exclusion of relevant articles, Zotero software [27] was employed for the management of the study references.

### Selection criteria and data extraction

The PECOS strategy guided the selection criteria as follows: Population, adult population over 18 years of age exposed to extreme vs. non-extreme temperature conditions; Exposure, individuals living in areas with high levels of greenness; Comparator, those exposed to low greenness; Outcome, mortality risk; and Study design, observational studies. The eligibility of studies for inclusion or exclusion was assessed independently by two reviewers.

The following data were extracted from the included studies: (1) authors and year of publication; (2) residence and region-country information; (3) study period; (4) extreme temperature data; (5) green space variable (The Normalized Difference Vegetation Index [NDVI], Enhanced Vegetation Index [EVI], % lack of green space, % lack of trees); and (6) risk ratios (RRs) with their lower and upper confidence intervals (CIs) for mortality, according to high and low greenness.

### Methodological quality assessment

The Quality Assessment tool for Observational Cohort and Cross-Sectional studies from the National Heart, Lung and Blood Institute [28] was utilized to ascertain the methodological quality of the included studies. The assessment tool includes 14 questions designed to evaluate a study’s internal validity by focusing on key concepts. A critical appraisal involved the evaluation of potential risks associated with selection bias, information bias, measurement bias, or confounding. These 14 criteria were used to assess whether the included studies clearly defined the research question and study population, employed valid and reliable exposure and outcome measures, and appropriately addressed potential confounding.

### Data analysis

This meta-analysis aimed to assess whether the association between extreme heat and mortality differed according to the level of greenness. To achieve this, two independent meta-analyses were conducted: one pooled the mortality RRs reported for areas with high greenness, and the other pooled those reported for areas with low greenness. The RRs and their corresponding 95% CIs were extracted from fully adjusted models. Given the expected variability among studies, random-effects models were applied [26] via the Sidik–Jonkman method [29]. The *I*^2^ statistic was used to quantify heterogeneity, which was categorized as not important (0–30%), moderate (30–50%), substantial (50–75%), or considerable (75–100%) [26]. The corresponding p values were also considered, with *P* < 0.05 indicating significant heterogeneity [30].

To formally compare the associations observed in low- and high-greenness areas, a ratio of RR (RoR) was calculated for each study by dividing the RR for low greenness by the corresponding RR for high greenness. The analysis was conducted on the logarithmic scale, with the log-RoR calculated as the difference between the log-transformed RRs for low and high greenness. Because both estimates were obtained from the same study and their within-study correlation was not reported, the variance of the log-RoR was calculated assuming a correlation coefficient of *r* = 0.50. Sensitivity analyses were conducted using alternative correlation coefficients of 0.25 and 0.75. The study-specific log-RoRs were pooled using a random-effects model with the Sidik–Jonkman estimator. Pooled estimates were subsequently exponentiated for interpretation, with RoR values greater than 1 indicating a stronger association between extreme heat and mortality in areas with low greenness than in areas with high greenness.

For the three studies that employed multiple measures of greenness [31–33], pooled RRs were calculated to assess the overall effect. This analysis was also conducted for the mortality results of one of the studies, which provided data on respiratory and cardiovascular mortality [34]. Given the potential dependencies in effect sizes, a multilevel meta-analytic approach was employed [35]. This method accounts for the hierarchical structure of the data, partitioning within- and between-study variance while adjusting for statistical dependencies [36]. By incorporating random effects at different levels, this approach enhances precision, reduces bias, and improves the robustness of the pooled effect estimates.

Subgroup analyses were conducted based on the continent of residence and the classification of the study populations as urban or urban–rural. The purpose of these analyses was to assess potential differences in effect size estimates according to geographic location and population density and to provide insight into the influence of environmental and contextual factors on the observed associations. Additional meta-regression analyses were considered but were not performed because potentially relevant study-level moderators, such as the greenness metric, spatial scale of exposure assessment, temperature metric, threshold used to define extreme heat, heat-exposure lag, study period, and level of covariate adjustment, were not consistently reported across the included studies. Consequently, these moderators were available for an insufficient number of studies, which could have resulted in statistically underpowered and potentially unreliable analyses [26].

Sensitivity analyses were performed to assess the robustness of the results. The leave-one-out method [37] was applied to determine the influence of each study on the overall estimates. Publication bias was assessed via Egger’s regression asymmetry test, and funnel plots were evaluated via visual inspection [38]. The software used for all the statistical analyses was R (Foundation for Statistical Computing, V.2023.12.1+402, Boston, USA) [39].

## Results

### Study selection

**Figure 1:**
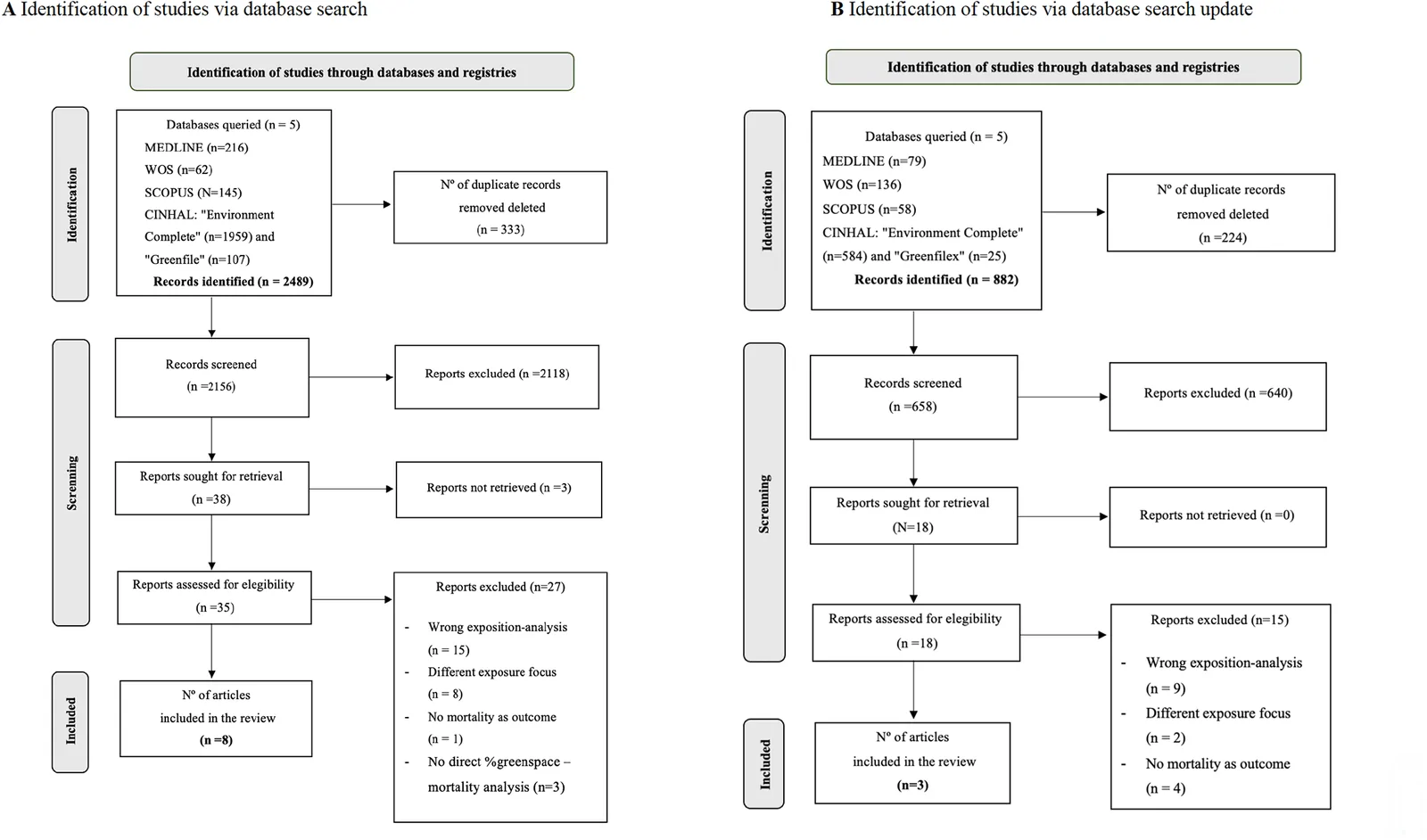
PRISMA 2020 flow diagram of study selection.

The systematic search yielded a total of 3371 articles. In the first stage, duplicate studies (557 articles) were eliminated. Of these, 53 were fully assessed for eligibility, and 42 were ultimately excluded (Table S2). Finally, a total of 11 [31–34, 40–46] studies were included in the systematic review and meta-analysis. The selection process for the articles is shown in Figure 1, according to the PRISMA guidelines [25].

### Baseline characteristics

The characteristics of the studies are summarized in Table 1. The studies were published between 2015 and 2025. In this review of observational studies, two were cohort studies, one prospective [45] and one retrospective [40]. Additionally, five were time-series studies [32, 33, 42, 44, 46], one was an ecological study [31], and three were case-crossover studies [34, 41, 43].

**Table 1:** Baseline characteristics of studies included in the systematic review and meta-analysis.

AUTHOR	COUNTRY	STUDY DESIGN	SAMPLE SIZE	AGE (YEARS)	GREEN MEASURE	EXPOSURE	MEANS	OUTCOMES	NUMBER OF DEATHS	RESULTS
**Choi et al. [31]**	Global	Ecological	452 locations (24 countries)	Population density	NDVI (0.53 ± 0.15) EVI (0.34 ± 0.10)	Follow-up: 2000–2018 (summer months) Heat exposure: 99th T percentile vs. MMT	Mean (SD) = 22.71ºC (4.42) Minimum 14.07ºC, maximum 30.18ºC	Daily mortality counts	19.3 deaths/day CVD mortality: 5 deaths/day and RD mortality: 2.2 deaths/day	Low greenness: RR 1.46 (1.31–1.62) High greeness: RR 1.19 (1.13–1.25) +20% greenness = −9.02% heat-attributable fraction
**Choi et al. [40]**	USA	Cohort	1275 census tracts in North Carolina	>20 years old Mean age: 72 years	NDVI (median 0.75) EVI (median 0.50)	Follow up: 2000–2016 (May–September) Heat-mortality risk: 99th T percentile vs. MMT	Mean T: 23.32°C (SD 3.86)	Heat-mortality risk	444,854 deaths	Low greenness: RR 1.08 (95% CI: 1.02–1.15) High greenness: RR 0.97 (95% CI: 0.87–1.08)
**Gronlund et al. [41]**	USA	Case-crossover	The 10 Michigan counties	>65 years old	Land use and presence of green spaces	Follow up: 1990–2007 (May–September) EH defined by the 97th and 99th 4-day T percentiles	Daily mean T for the study period	Deaths records	91.6 deaths/day	Low greenness: OR 1.17 (1.06–1.29) High greenness: OR 0.98 (0.89–1.07)
**Hu et al. [46]**	China	Case time-series design	1085 subdistricts	NA	Green and Blue Space Exposure Index	Follow-up: 2009–2020 (warm season; annual daily data)	Heat defined by high T: P95th vs. MMT	All-cause mortality	241,640 deaths/year	Low greenness: RR1.18 (95% CI:1.16–1.21) High greenness: RR1.13 (95% CI: 1.12–1.15)
**Luque-Garcia et al. [42]**	Jordan	Time series	42 districts	Population-based	NDVI (low ≤0.173 vs. high >0.173)	Follow-up: 2000–2020 (May–September); MMT (22.20°C) vs. P99th (32.28°C)	T Max: 32.28°C (29.79–35.02°C)	Daily all-cause mortality	171,284 deaths	Low greenness: RR 1.39 (95% CI: 1.22–1.58) High greenness: RR 1.28 (95% CI: 1.13–1.45)
**Pascal et al. [32]**	France	Time series	Paris (143 communes) vs. Grande Couronne (1157 communes)	≥75 years: 5.4% population	Lack of green space (2006) and tree cover (2015)	Follow-up: 1981–2010; heat waves defined as ≥3 consecutive days with mean T above the P97.5th and at least one day above the P99.5th during summer	Paris: Mean T = 18.6°C; P99.5th = 32.5°C Grande Couronne: Mean T = 18.4°C; P99.5th = 31.4°C	Daily all-cause mortality by municipality	601,643 deaths	Paris: RR 1.94 (95% CI: 1.82–2.06) for lack of green space; RR 1.94 (95% CI: 1.82–2.07) for lack of trees. Grande Couronne: RR 1.49 for lack of green space and 1.39 for lack of trees
**Qiu et al. [43]**	China	Case-crossover	21,775 habitants	>65 years old (x = 75 years old)	Follow up: 2000–2014 NDVI (250 m around participants’ residences)	Follow up: 2000–2014 Heat-mortality risk: P95th vs. P50th T percentile	High T: P95th = 44°C	Individual mortality	NR	Low greenness: RR 1.38 (95% CI: 0.79–2.42) High greenness: RR 1.06 (95% CI: 0.85–1.31)
**Song et al. [33]**	China	Case time series	286 districts in Hong Kong	Population deaths in districts. Subgroups by age (0–64 and ≥65 years)	Follow up: 2006–2018 NDVI, green space and eye-level street greenery	Follow-up: 2005–2018 (June–October) Heat-mortality risk: P99th vs. MMT	Mean T: 28.0°C; EH: P99th = 30.9°C	Summer mortality (June–October)	213,505 deaths	Urban areas (Low vegetation): RR 9.6 (95% CI: 3.5–16.1) High vegetation: RR 1.5 (95% CI: 0.6–2.4)
**Wicki et al. [44]**	Switzerland	Time series	8 major cities in Swiss	Deaths records in 8 Swiss cities	2014 NDVI data (500 m around participants’ geocoded residences)	Follow-up: 2003–2016 (May–September) UHI identified using the hottest 1% of days in each city	Mean T: 23.4°C; P99th: 35°C	Warm-season mortality (May–September)	53,593 deaths	Low greenness: RR 1.13 (95% CI: 0.89–1.44) High greenness: RR 1.28 (95% CI: 1.17–1.40)
**Zhang et al. [45]**	China	Cohort	34,394 participants in 452 locations	≥65 years (mean 91.06 ± 9.65)	Annual NDVI (2000–2014) Mean NDVI 0.41 ± 0.13	Follow-up: 2000–2014 (average daily T during the warmest months in each city)	Heat waves: ≥3 consecutive days with Tmax ≥ P92.5th during the warmest months	Mortality records of the cities	HR and 95% CI for each 3-day increase in heat waves and mortality at different NDVI levels	Low greenness: RR = 1.46 (95% CI: 1.31, 1.62) High greenness: RR = 1.19 (95% CI: 1.13, 1.25) CI: 1.13, 1.25).
**Zhang et al. [34]**	Germany	Case-crossover	380 districts	Not specified (district level)	Urbanized areas and green areas	Follow-up: 2000–2016 (warm season: May–September)	Heat-related mortality: Greenness P25th vs. P75th	CVD and RD mortality	2,050,764 (CVD deaths) and 299,249 (RD deaths)	CVD: RR Low greenness 1.27 (95% CI: 1.25–1.29) vs. High greenness 1.21 (95% CI: 1.20–1.23) RD: RR Low greenness 1.40 (95% CI: 1.36–1.45) vs. High greenness 1.27 (95% CI: 1.23–1.32)

Abbreviations: CI (Confidence Interval), CVD (Cardiovascular Disease), EH (Extreme Heat), EVI (Enhanced Vegetation Index), HR (Hazard Ratio), Max (Maximum), Min (Minimum), MMT (Minimum Mortality Temperature), NDVI (Normalized Difference Vegetation Index), OR (Odds Ratio), RD (Respiratory Disease), RR (Relative Risk), T (Temperature).

Among the articles reviewed, five were carried out in the Asian context (China [33, 43, 45, 46] and Jordan [42]), two were conducted in the United States [40, 41]), three were performed in Europe (France [32]; Switzerland [44], and Germany [34]), and one was conducted on a global scale [31]. Studies have covered long timeframes, such as 1981–2010 [32] and 2000–2018 [31].

### Population

Several studies have focused on the general population [32–34, 40, 42, 44], while others have focused on older adults (over 65 years) [41, 43, 45–47]. In addition, certain studies analyzed participants with specific health conditions, such as preexisting cardiovascular or respiratory diseases [31, 34].

The weather data on temperature were obtained from various sources, such as local weather stations [31–34, 44, 46], or were provided by environmental agencies or climatological departments [41–43, 45]. The definitions of extreme heat and heat waves have varied across studies, with temperature thresholds based on percentiles (90th, 95th, or 99th) [31–34, 40, 41, 43, 45, 46]. Heat waves are generally defined as periods of three or more days with mean temperatures above the 97.5th percentile, including at least one day above the 99.5th percentile [32]. The study periods varied, with a primary focus on the warmest season [31–33, 40–42, 44, 45], and other studies measured continuous daily average temperatures throughout the entire study period [34, 43].

### Exposure

The greenness was measured mostly by the NDVI [42–45]. Some studies focused on specific areas close to participants’ homes [43–45], whereas others were based on the distribution of the NDVI within each geographic study area [42]. Furthermore, one study combined the NDVI with the EVI [40]. Other studies have employed alternative measures, such as the percentage coverage of urbanized areas and green areas [34] or the percentage of “nongreen space” [41]. Other studies have combined various measures [29–31, 45, 46]. For example, the density and distribution of green space, with a particular focus on areas where there is a lack of green spaces and trees [32], as well as the percentage of green space and eye-level street greenness [38].

### Outcomes

The primary outcome of interest in all the studies was mortality related to extreme heat events. Several studies have utilized official death records from governmental health authorities [40, 44–46]. These records provide standardized data on all deaths, including causes and geographic information. Additionally, some studies [32, 34, 41] used daily mortality data to assess the effects of heat, enabling comprehensive analysis of heat-related mortality across diverse locations. In addition, two studies [31, 33] collected mortality data during specific periods to assess the effects of heat. Conversely, Luque et al. [48] utilized time series analysis to evaluate temperature-related mortality.

### Methodological quality

Eight studies [31–34, 40, 42, 45, 46] were classified as good quality, and three studies [41, 43, 44] were classified as moderate quality. The most common limitations across studies were the absence of outcome assessor blinding to exposure status, inadequate sample size, and limited assessment of exposure, which was typically conducted once (Table S3).

#### Meta-analysis

**Figure 2:**
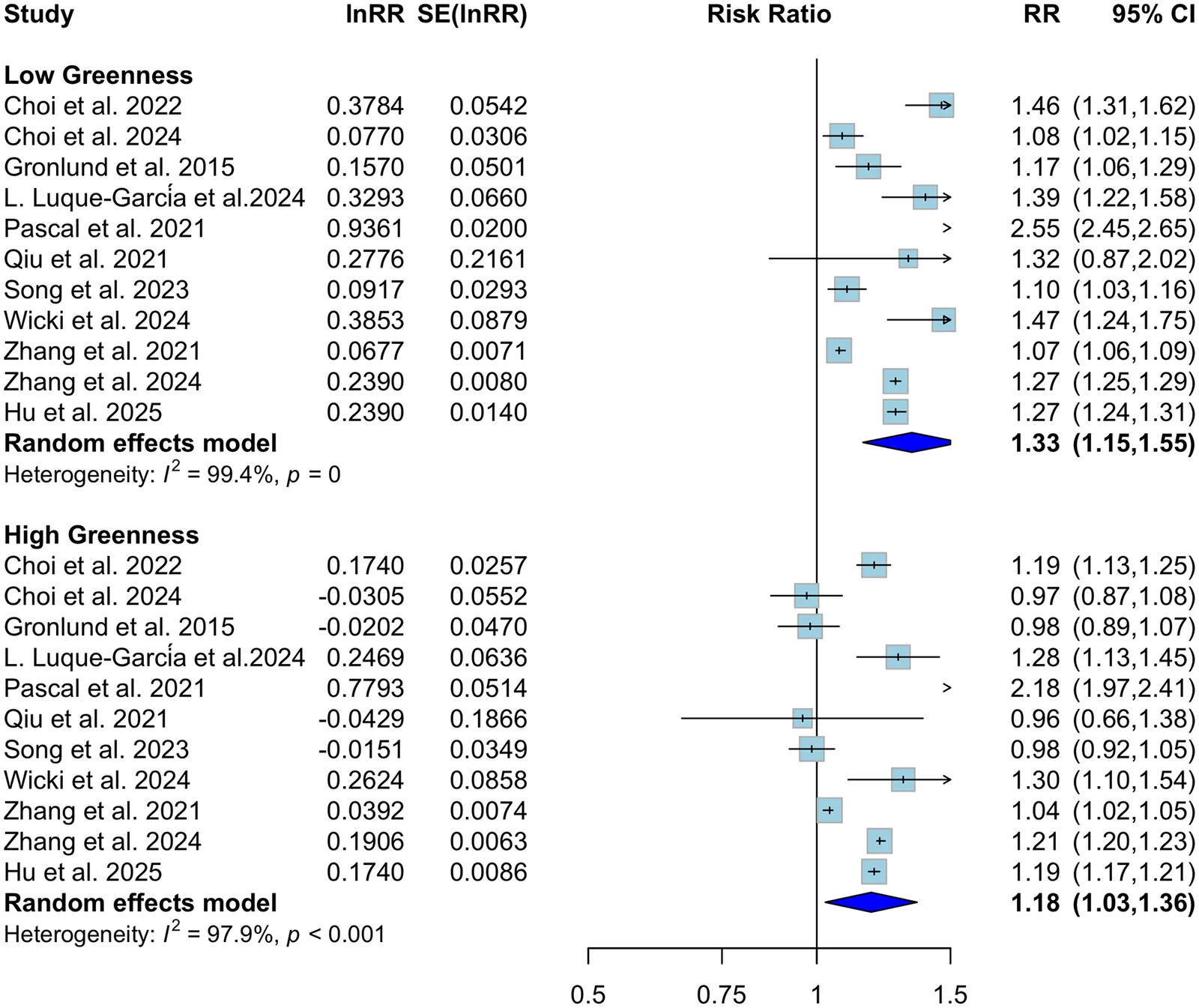
Forest plot of meta-analysis results of greenness exposure and mortality during extreme heat. The upper panel shows the pooled effect estimates for populations with low greenness exposure, while the lower panel represents those with high greenness exposure. Each square denotes the effect estimate of an individual study, with horizontal lines indicating 95% confidence intervals. Abbreviations: CI, Confidence Interval; RR, Relative Risk; SE, Standard Error.

The results of the meta-analysis are displayed in Figure 2. Extreme heat was associated with a significantly increased risk of mortality in both low- and high-greenness areas. The pooled RR for areas exhibiting the lowest levels of greenness was 1.33 (95% CI: 1.15–1.55; *I*^2^ = 99.4%) in areas with the lowest levels of greenness and 1.18 (95% CI: 1.03–1.36; *I*^2^ = 97.9%) in areas with the highest levels of greenness. The pooled RoR showed that the association between extreme heat and mortality was significantly stronger in low-greenness areas than in high-greenness areas (RoR = 1.11; 95% CI: 1.06–1.15; *I*^2^ = 90.5%), indicating an 11% stronger heat–mortality association in low-greenness areas. Sensitivity analyses using alternative assumptions for the within-study correlation yielded similar pooled estimates. The pooled RoR was 1.10 (95% CI: 1.06–1.15; *I*^2^ = 85.5%) when a correlation of 0.25 was assumed and 1.11 (95% CI: 1.07–1.16; *I*^2^ = 95.5%) when a correlation of 0.75 was assumed. These findings indicate that the pooled RoR was robust across the different assumptions regarding the within-study correlation, although heterogeneity remained considerable in all analyses.

### Subgroup analyses

#### Geographical location by continent

The results of the subgroup analyses according to geographical region are displayed in Table 2. In the American subgroup, which included only studies conducted in the USA, the pooled RR for low greenness was 1.25 (95% CI: 0.94–1.67; *I*^2^ = 95.7%). In Asian studies, the corresponding pooled RR was 1.19 (95% CI: 1.08–1.32; *I*^2^ = 97.0%), whereas the largest pooled RR was observed in European studies (RR = 1.69; 95% CI: 1.11–2.56; *I*^2^ = 99.8%). For areas with high greenness, the pooled RR was 1.61 (95% CI: 0.89–2.90; *I*^2^ = 99.1%) in the USA, 1.16 (95% CI: 1.05–1.29; *I*^2^ = 97.4%) in Asia, and 1.43 (95% CI: 0.93–2.20; *I*^2^ = 99.0%) in Europe. No statistically significant differences in the pooled associations were observed across geographical regions (Table 2).

**Table 2:** Subgroup analyses by continent and study setting.

BY CONTINENT						
GREENNESS LEVEL	CONTINENT	*N*	ESTIMATE (95% CI)	*P* VALUE (SUBGROUP)	*I*^2^ (%)	*P* VALUE (HETEROGENEITY)
Low	America	*n* = 2	1.251 (0.936–1.672)	0.2849	95.7%	<0.0001
	Asia	*n* = 5	1.193 (1.078–1.321)		97.0%	<0.0001
	Europe	*n* = 3	1.686 (1.110–2.561)		99.8%	<0.0001
High	America	*n* = 2	1.608 (0.891–2.903)	0.3880	99.1%	<0.0001
	Asia	*n* = 5	1.163 (1.048–1.291)		97.4%	<0.0001
	Europe	*n* = 3	1.432 (0.931–2.203)		99.0%	<0.0001
**BY STUDY SETTING**						
**GREENNESS LEVEL**	**SETTING**	***N***	**ESTIMATE (95% CI)**	***P* VALUE (SUBGROUP)**	***I*^2^ (%)**	***P* VALUE (HETEROGENEITY)**
Low	Urban	*n* = 4	1.274 (1.100–1.476)	0.6159	89.3%	<0.0001
	Mixed (urban-rural)	*n* = 7	1.365 (1.091–1.706)		99.7%	<0.0001
High	Urban	*n* = 4	1.366 (0.995–1.876)	0.5747	98.7%	<0.0001
	Mixed (urban-rural)	*n* = 7	1.225 (0.995–1.508)		98.6%	<0.0001

*n* represents the number of studies included in each analysis. The American subgroup included only studies conducted in the USA.

Abbreviations: CI (Confidence Interval), *I*^2^ (Heterogeneity Statistic).

#### Study setting

In studies conducted in urban settings, the pooled RR was 1.27 (95% CI: 1.10–1.48; *I*^2^ = 89.3%) for areas with low greenness and 1.37 (95% CI: 0.99–1.88; *I*^2^ = 98.7%) for areas with high greenness. In studies including mixed urban and rural settings, the corresponding pooled RRs were 1.36 (95% CI: 1.09–1.71; *I*^2^ = 99.7%) and 1.23 (95% CI: 0.99–1.51; *I*^2^ = 98.6%), respectively. No statistically significant differences in the pooled associations were observed between study settings (Table 2).

### Sensitivity analysis and publication bias

The leave-one-out sensitivity analyses showed that both the pooled effect estimates and the levels of heterogeneity remained similar after the exclusion of each individual study (Figures S1–S2). According to the Egger test and the funnel plot asymmetry (Table S4 and Figure S3), Egger's regression indicated no evidence of publication bias in either the low-greenness analysis (*P* = 0.41) or the high-greenness analysis (*P* = 0.84).

## Discussion

This systematic review and meta-analysis aimed to examine whether the association between extreme heat and mortality differed according to the level of greenness. Extreme heat was associated with increased mortality in both low- and high-greenness areas. However, the pooled RoR indicated that the heat–mortality association was 11% stronger in low-greenness areas than in high-greenness areas, suggesting that greater greenness may attenuate vulnerability to extreme heat. Nevertheless, these findings should be interpreted cautiously because of the limited number of included studies and the considerable between-study heterogeneity.

The results of our meta-analysis are consistent with the findings of previous systematic reviews. These studies indicated that heat waves and high temperatures have significant impacts on public health, with increased mortality rates [49, 50]. In addition, a further meta-analysis suggested that heat waves are significantly associated with increased mortality, with an RR of 1.19 for non-accidental deaths (95% CI: 1.13–1.27) [51]. Our findings are consistent with this evidence, indicating an increased mortality risk for those exposed to extreme temperatures and heat waves compared with those exposed to lower temperature percentiles. A study revealed that, from 2000 to 2019, approximately 489,000 heat-related deaths occurred annually, including more than 70,000 deaths during the 2003 European heat wave [52]. In view of the inevitable warming trend in the next few decades, the mortality burden associated with heat exposure is expected to increase [53].

Most health effects directly attributable to rising temperatures can be mitigated through the implementation of strategies aimed at reducing emissions and limiting global warming [54–56]. In this context, exposure to higher levels of greenness emerges as a valuable nature-based adaptation strategy [57, 58]. Some types of green spaces, such as tree-covered areas, have been inversely associated with heat-related mortality, highlighting their potential role in mitigating the health impacts of extreme urban heat [59, 60]. Consistent with this, previous systematic reviews have examined the relationship between vegetation and reduced mortality [18, 23]. Specifically, Rojas-Rueda et al. [23] examined the relationship between residential exposure to green space and all-cause mortality, reporting a hazard ratio of 0.96 (95% CI: 0.94–0.97) for all-cause mortality associated with each 0.1 unit increase in the NDVI, suggesting a protective effect of green spaces on mortality. While the previous review focused on all-cause mortality, our systematic review and meta-analysis specifically address heat-related mortality, which is becoming important in the context of climate change and more frequent extreme heat events. By focusing on heat-related mortality, we can provide a more precise understanding of environmental vulnerabilities. Our findings suggest that people living in greener areas may be less vulnerable to the health impacts of extreme heat. These findings reinforce the idea that the integration and preservation of green spaces may represent an effective measure to mitigate the effects of extreme heat on human mortality [61–63].

The subgroup analysis showed that the pooled associations varied descriptively across geographical regions. In Europe, America, and Asia, the pooled RRs were numerically lower in areas with higher greenness than in those with lower greenness. However, these patterns should be interpreted cautiously because some geographical subgroups included only two or three studies, no statistically significant differences were observed between regions, and substantial heterogeneity persisted within most subgroups. The largest pooled estimates were observed among the European studies. This pattern may reflect differences in urban form, population density, climatic conditions, green infrastructure, population vulnerability, and the implementation of local heat-adaptation strategies. For example, populations in regions historically characterized by milder climates may be less adapted to increasingly frequent episodes of extreme heat [64, 65]. In America, previous evidence suggests that urban greening may help reduce heat exposure and improve air quality and, when combined with emergency response plans and resilient urban infrastructure, may contribute to reducing mortality during extreme heat events [66, 67]. Nevertheless, given the limited number of studies within each region and the considerable residual heterogeneity, these potential explanations remain speculative. The significant associations between low greenness and heat-related mortality observed in all the geographical regions analyzed suggest that limited vegetation may contribute to greater vulnerability to extreme heat, although the observational evidence does not establish a causal relationship.

The issue of regional disparities in vulnerability to extreme heat is a growing concern in climate adaptation research. These disparities could be attributed to a variety of interrelated factors, including urban design [68], population density [69], and the efficacy of local heat-adaptation strategies [70, 71]. In this context, the lack of studies on heat-related mortality in Africa and Oceania in this analysis highlights an important geographical evidence gap. This paucity of research and the limited attention paid to inequalities in the response to heat risks are particularly concerning [8, 72, 73]. Limited green infrastructure and reduced capacity to implement effective heat-adaptation strategies may further increase vulnerability in underserved regions. Recent studies have demonstrated that access to green spaces is unequal, with more equitable access in cities in the Global North and limited access in the Global South, especially for vulnerable populations [74]. This disparity may contribute to increased vulnerability to heat-related mortality, emphasizing the need to address these inequalities in future climate resilience strategies [75].

The pooled associations also varied descriptively by study setting. In both urban and mixed urban–rural settings, the pooled RRs were numerically lower in high-greenness areas than in low-greenness areas. However, no statistically significant differences were observed between study settings, and considerable heterogeneity persisted within the subgroups, suggesting that the association between greenness and heat-related mortality may also be influenced by local environmental and sociospatial conditions. These results underscore the necessity of approaching heat resilience and adaptation planning from a holistic perspective that considers local environmental and sociospatial conditions. In urban areas, for example, the urban heat island effect exacerbates heat-related challenges, making green spaces a potentially important component of adaptation strategies [16, 61, 71, 76]. For example, one of the key strategies employed in a study in Melbourne was the promotion of shade tree planting in areas of high pedestrian activity, which resulted in significant localized cooling effects [77]. Conversely, rural areas may benefit from different mechanisms, such as natural cooling and lower population concentrations, which could contribute to local heat resilience [78, 79]. Research in Spain suggests that some rural areas may be better able to cope with extreme heat because of their higher levels of natural vegetation [80]. Overall, these findings highlight the importance of adapting green-infrastructure and heat-resilience strategies to the characteristics of each local context in a warming world.

The present systematic review and meta-analysis have several limitations that should be addressed. A key limitation is the significant between-study heterogeneity observed in most pooled estimates. Subgroup analyses according to continent and study setting were conducted to explore potential sources of variability; however, considerable heterogeneity persisted within most subgroups, indicating that these factors did not fully explain the differences across studies. Additional meta-regression analyses were not considered statistically reliable because potentially relevant characteristics were inconsistently reported. The remaining heterogeneity may reflect differences in study design, population characteristics, climatic and geographical conditions, definitions of extreme heat and heat-related mortality, greenness metrics, assessment periods, spatial resolution, and covariate adjustment. Consequently, the pooled estimates represent an average across heterogeneous settings and should not be assumed to apply uniformly to all populations and geographical contexts. In addition, most included studies were conducted in high-income countries, limiting the generalizability of the findings to other settings, particularly in low- and middle-income regions where exposure patterns and adaptive capacity may differ substantially. Furthermore, residual confounding remains possible despite adjustment for key variables, and temporal mismatches between exposure and outcome measurements may have influenced the accuracy of the estimates. Taken together, the substantial between-study heterogeneity, limited geographical representation, and observational nature of the included evidence warrant cautious interpretation of the findings. Future research should prioritize standardized exposure and outcome definitions, consistent analytical approaches, and broader geographical representation to improve comparability and reduce unexplained variability across studies.

Conversely, this study has several strengths that should be mentioned. To the best of our knowledge, this is the first meta-analysis to examine the association between greenness and mortality during periods of extreme heat using observational studies. Moreover, the subgroup analyses conducted by geographical region and study setting provide a more nuanced and detailed understanding of the results. This review also identifies important regional evidence gaps, particularly in Africa and Oceania, highlighting the need for future research in these regions. Advancing robust research efforts in these regions is crucial to improving our understanding of the complex associations between heat exposure and mortality and informing the development of effective, locally tailored public health responses. The results of this systematic review and meta-analysis suggest that greenness could play an important role in mitigating heat-related risks to human health.

## Conclusions

In conclusion, higher temperatures were associated with increased mortality in both low- and high-greenness areas. However, the heat–mortality association was approximately 11% stronger in low-greenness areas than in high-greenness areas, suggesting that greater greenness may attenuate vulnerability to extreme heat. Nevertheless, the substantial heterogeneity, possible small-study effects, and observational nature of the included evidence warrant cautious interpretation. These findings may inform public health and climate-adaptation strategies, particularly those addressing populations and regions that are especially vulnerable to extreme heat. Local authorities may consider the promotion and preservation of urban green spaces as part of broader, context-specific heat-adaptation strategies. Further high-quality research is needed to determine whether and to what extent different types, levels, and spatial configurations of greenness modify heat-related mortality and to identify the most effective green-infrastructure strategies across diverse urban, rural, climatic, and geographical settings. Such evidence will be important for strengthening climate resilience and informing public health responses to global warming.

## Data Availability

All data analyzed in this systematic review and meta-analysis were obtained from published peer-reviewed studies and are presented within the article and its supplementary materials. Further information may be obtained from the corresponding author upon reasonable request.

## Additional Files

The additional files for this article can be found as follows:

Supplementary Table S1. PRISMA Checklist 2020.

Supplementary Table S2. List of studies fully assessed for eligibility and excluded.

Supplementary Table S3. Methodological quality assessment of included studies.

Supplementary Table S4. Meta-bias for the cross-sectional associations between greenness and mortality during extreme heat periods.

Supplementary Figure S1. Leave-one-out analysis of the association between low greenness exposure and mortality during extreme heat periods.

Supplementary Figure S2. Leave-one-out analysis of the association between high greenness exposure and mortality during extreme heat periods.

Supplementary Figure S3. Publication bias assessment.

## References

[r1] Intergovernmental Panel on Climate Change. Summary for Policymakers: Climate Change 2023: Synthesis Report. IPCC; 2023. Accessed November 8, 2024. https://www.ipcc.ch/report/ar6/syr/downloads/report/IPCC_AR6_SYR_SPM.pdf.

[r2] Ebi KL, Capon A, Berry P, et al. Hot weather and heat extremes: Health risks. Lancet. 2021;398(10301):698–708. doi:10.1016/S0140-6736(21)01208-3. 34419205

[r3] Ebi KL, Vanos J, Baldwin JW, et al. Extreme weather and climate change: Population health and health system implications. Annu Rev Public Health. 2021;42:293–315. 10.1146/annurev-publhealth-012420-105026 PMC9013542 33406378

[r4] World Meteorological Organization. Heatwave [Internet]. Geneva: WMO. https://wmo.int/topics/heatwave.

[r5] Hansen J, Sato M, Ruedy R. Perception of climate change. Proc Natl Acad Sci U S A. 2012;109(37):E2415–E2423. 10.1073/pnas.1205276109 PMC3443154 22869707

[r6] World Meteorological Organization. WMO Global Annual to Decadal Climate Update 2023 [Internet]. Geneva: World Meteorological Organization; 2023. https://library.wmo.int/records/item/66224-wmo-global-annual-to-decadal-climate-update.

[r7] Gasparrini A, Guo Y, Hashizume M, et al. Mortality risk attributable to high and low ambient temperature: A multicountry observational study. Lancet. 2015;386(9991):1085–1092. 10.1016/S0140-6736(14)62114-0 PMC4521077 26003380

[r8] Romanello M, Walawender M, Hsu SC, et al. The 2024 report of the Lancet Countdown on health and climate change: Facing record-breaking threats from delayed action. Lancet. 2024;404(10465):1847–1896. 10.1016/S0140-6736(24)01822-1 PMC7616816 39488222

[r9] Centers for Disease Control and Prevention. Climate and Health: Extreme Heat [Internet]. Atlanta: CDC; 2021. https://www.cdc.gov/climate-health/php/effects/temperature-extremes.html.

[r10] García-León D, Masselot P, Mistry MN, et al. Temperature-related mortality burden and projected change in 1368 European regions: A modelling study. Lancet Public Health. 2024;9(9):e644–e653. doi:10.1016/S2468-2667(24)00179-8. Epub 2024 Aug 21. PMID: 39181156.

[r11] Álvarez-Miño L, Taboada-Montoya R. Efectos del cambio climático en la salud pública, 2015–2020. Una revisión sistemática. Rev Esp Salud Publica. 2021;95:e202103042. 33729215

[r12] Ferrer M, Poljanšek K, Clark I, De Groeve T, eds. Science for Disaster Risk Management 2017: Knowing Better and Losing Less [Internet]. Luxembourg: Publications Office of the European Union; 2017.

[r13] Liu J, Varghese BM, Hansen A, et al. Heat exposure and cardiovascular health outcomes: A systematic review and meta-analysis. Lancet Planet Health. 2022;6(6):e484–e495. doi:10.1016/S2542-5196(22)00117-6. PMID: 35709806.

[r14] Royé D, Codesido R, Tobías A, Taracido M. Heat wave intensity and daily mortality in four of the largest cities of Spain. Environ Res. 2020;182:109027. 10.1016/j.envres.2019.109027 31884190

[r15] Guirguis K, Gershunov A, Tardy A, Basu R. The impact of recent heat waves on human health in California. J Appl Meteorol Climatol. 2014;53:3–19. doi:10.1175/JAMC-D-13-0130.1.

[r16] Ho JY, Shi Y, Lau KKL, Ng EYY, Ren C, Goggins WB. Urban heat island effect-related mortality under extreme heat and non-extreme heat scenarios: A 2010–2019 case study in Hong Kong. Sci Total Environ. 2023;858(pt 1):159791. doi:10.1016/j.scitotenv.2022.159791. Epub 2022 Nov 1. PMID: 36328261.

[r17] Sharma R, Hooyberghs H, Lauwaet D, De Ridder K. Urban heat island and future climate change: Implications for Delhi's heat. J Urban Health. 2019;96(2):235–251. doi:10.1007/s11524-018-0322-y. PMID: 30353483; PMCID: . PMC6458210

[r18] Gascon M, Triguero-Mas M, Martínez D, et al. Residential green spaces and mortality: A systematic review. Environ Int. 2016;86:60–67. doi:10.1016/j.envint.2015.10.013. Epub 2015 Nov 2. PMID: 26540085.

[r19] Chi D, Manoli G, Lin B, et al. Residential tree canopy configuration and mortality in 6 million Swiss adults: A longitudinal study. Lancet Planet Health. 2025;9(3):e186–e195. doi:10.1016/S2542-5196(25)00022-1. 40120625

[r20] Bianconi A, Longo G, Coa AA, Fiore M, Gori D. Impacts of urban green on cardiovascular and cerebrovascular diseases: A systematic review and meta-analysis. Int J Environ Res Public Health. 2023;20(11):5966. doi:10.3390/ijerph20115966. PMID: 37297570; PMCID: . PMC10253108

[r21] Bowler DE, Buyung-Ali LM, Knight TM, Pullin AS. A systematic review of evidence for the added benefits to health of exposure to natural environments. BMC Public Health. 2010;10:456. doi:10.1186/1471-2458-10-456. PMID: 20684754; PMCID: . PMC2924288

[r22] Barboza EP, Cirach M, Khomenko S, et al. Green space and mortality in European cities: A health impact assessment study. Lancet Planet Health. 2021;5(10):e718–e730. doi:10.1016/S2542-5196(21)00229-1. 34627476

[r23] Rojas-Rueda D, Nieuwenhuijsen MJ, Gascon M, Perez-Leon D, Mudu P. Green spaces and mortality: A systematic review and meta-analysis of cohort studies. *Lancet Planet Health*. Erratum in: Lancet Planet Health. 2019;3(11):(8):e469–e477, e504. doi:10.1016/S2542-5196(19)30215-3. Erratum in: Lancet Planet Health. 2021;5(8):e504. doi:10.1016/S2542-5196(21)00208-4. PMID: 31777338; PMCID: . PMC6873641

[r24] Kua KP, Lee S. The influence of residential greenness on mortality in the Asia-Pacific region: A systematic review and meta-analysis. Perspect Public Health. 2021;141(6):342–353. 10.1177/17579139211011496 34120524

[r25] Page MJ, McKenzie JE, Bossuyt PM, et al. A declaração PRISMA 2020: Diretriz atualizada para relatar revisões sistemáticas. Rev Panam Salud Publica. 2022;46:e112. doi:10.26633/RPSP.2022.112. PMID: 36601438; PMCID: . PMC9798848

[r26] Higgins J, Green S, eds. Cochrane Handbook for Systematic Reviews of Interventions. Cochrane; 2011. https://training.cochrane.org/handbook.

[r27] Ahmed KKM, Al Dhubaib BE. Zotero: A bibliographic assistant to researcher. J Pharmacol Pharmacother. 2011;2(4):303–305. 10.4103/0976-500X.85940 PMC3198533 22025866

[r28] National Heart, Lung, and Blood Institute. Study Quality Assessment Tools [Internet]. Bethesda (MD): National Institutes of Health. https://www.nhlbi.nih.gov/health-topics/study-quality-assessment-tool.

[r29] Sidik K, Jonkman JN. A note on variance estimation in random effects meta-regression. J Biopharm Stat. 2005;15(5):823–838. 10.1081/BIP-200067915 16078388

[r30] Higgins JPT, Thompson SG. Quantifying heterogeneity in a meta-analysis. Stat Med. 2002;21(11):1539–1558. 10.1002/sim.1186 12111919

[r31] Choi HM, Lee W, Royé D, et al. Effect modification of greenness on the association between heat and mortality: A multi-city multi-country study. EBioMedicine. 2022;84:104251. doi:10.1016/j.ebiom.2022.104251. PMC9471476 36088684

[r32] Pascal M, Goria S, Wagner V, et al. Greening is a promising but likely insufficient adaptation strategy to limit the health impacts of extreme heat. Environ Int. 2021;151:106441. doi:10.1016/j.envint.2021.106441. Epub 2021 Feb 25. PMID: 33640693.

[r33] Song J, Gasparrini A, Fischer T, Hu K, Lu Y. Effect modifications of overhead-view and eye-level urban greenery on heat–mortality associations: Small-area analyses using case time series design and different greenery measurements. Environ Health Perspect. 2023;131(9):097007. 10.1289/EHP12589 PMC10510815 37728899

[r34] Zhang S, Breitner S, de'Donato F, et al. Heat and cause-specific cardiopulmonary mortality in Germany: A case-crossover study using small-area assessment. Lancet Reg Health Eur. 2024;46:101062. 10.1016/j.lanepe.2024.101049 PMC11406445 39290807

[r35] Cheung MW-L. Modeling dependent effect sizes with three-level meta-analyses: A structural equation modeling approach. Psychol Methods. 2014;19(2):211–229. 10.1037/a0032968 23834422

[r36] Van den Noortgate W, López-López JA, Marín-Martínez F, Sánchez-Meca J. Three-level meta-analysis of dependent effect sizes. Behav Res Methods. 2013;45(2):576–594. 10.3758/s13428-012-0261-6 23055166

[r37] Jeong U, Han SH, Kim D, Kim S, Cheong EJ. Exploring the efficient irrigation period for *Larix kaempferi* seedlings in nursery pots in greenhouse conditions using optical measurements. Forests. 2024;15(8):1303.

[r38] Sterne JAC, Egger M, Smith GD. Systematic reviews in health care: Investigating and dealing with publication and other biases in meta-analysis. BMJ. 2001;323(7304):101–105. 10.1136/bmj.323.7304.101 PMC1120714 11451790

[r39] R Core Team. R: A Language and Environment for Statistical Computing. R Foundation for Statistical Computing; 2020.

[r40] Choi HM, Heo S, Bell ML. The effect modification of greenspace and impervious surface on the heat-mortality association: Differences by the dissimilarity index. Sci Total Environ. 2024;908:168074. 10.1016/j.scitotenv.2023.168074 PMC10841598 37898198

[r41] Gronlund CJ, Berrocal VJ, White-Newsome JL, Conlon KC, O'Neill MS. Vulnerability to extreme heat by socio-demographic characteristics and area green space among the elderly in Michigan, 1990–2007. Environ Res. 2015;136:449–461. 10.1016/j.envres.2014.08.042 PMC4282170 25460667

[r42] Luque-Garcia L, Bataineh S, Al-Bakri J, Abdulla FA, Al-Delaimy WK. The heat-mortality association in Jordan: Effect modification by greenness, population density and urbanization level. Sci Total Environ. 2024;952:175841. 10.1016/j.scitotenv.2024.176010 PMC13377650 39233083

[r43] Qiu C, Ji JS, Bell ML. Effect modification of greenness on temperature-mortality relationship among older adults: A case-crossover study in China. Environ Res. 2021;197:111112. 10.1016/j.envres.2021.111112 PMC8343965 33838131

[r44] Wicki B, Flückiger B, Vienneau D, de Hoogh K, Röösli M, Ragettli MS. Socio-environmental modifiers of heat-related mortality in eight Swiss cities: A case time series analysis. Environ Res. 2024;246:118116. 10.1016/j.envres.2024.118116 38184064

[r45] Zhang H, Liu L, Zeng Y, Liu M, Bi J, Ji JS. Effect of heatwaves and greenness on mortality among Chinese older adults. Environ Pollut. 2021;290:118009. 10.1016/j.envpol.2021.118009 34523521

[r46] Hu K, Wang S, Fei F, et al. Blue space scenarios in China. Environ Health Perspect. 2025;133(5):EHP14014. doi:10.1289/EHP14014. PMC12097534 40193182

[r47] Burkart K, Meier F, Schneider A, et al. Modification of heat-related mortality in an elderly urban population by vegetation (urban green) and proximity to water (urban blue): Evidence from Lisbon, Portugal. Environ Health Perspect. 2016;124(7):927–934. 10.1289/ehp.1409529 PMC4937850 26566198

[r48] Luque-García L, Bataineh S, Al-Bakri J, Abdulla FA, Al-Delaimy WK. The heat-mortality association in Jordan: Effect modification by greenness, population density and urbanization level. Sci Total Environ. 2024;952:175841. 10.1016/j.scitotenv.2024.176010 PMC13377650 39233083

[r49] Sun Z, Chen C, Xu D, Li T. Effects of ambient temperature on myocardial infarction: A systematic review and meta-analysis. Environ Pollut. 2018;241:1106–1114. 10.1016/j.envpol.2018.06.045 30029319

[r50] Moon J. The effect of the heatwave on the morbidity and mortality of diabetes patients: A meta-analysis for the era of the climate crisis. Environ Res. 2021;195:110762. 10.1016/j.envres.2021.110762 33515577

[r51] Pan R, Xie M, Chen M, Zhang Y, Ma J, Zhou J. The impact of heat waves on the mortality of Chinese population: A systematic review and meta-analysis. Medicine (Baltimore). 2023;102(13):e33345. 10.1097/MD.0000000000033345 PMC10063284 37000079

[r52] Zhao Q, Guo Y, Ye T, et al. Global, regional, and national burden of mortality associated with non-optimal ambient temperatures from 2000 to 2019: A three-stage modelling study. Lancet Planet Health. 2021;5(7):e415–e425. 10.1016/S2542-5196(21)00081-4 34245712

[r53] Gasparrini A, Guo Y, Sera F, et al. Projections of temperature-related excess mortality under climate change scenarios. Lancet Planet Health. 2017;1(9):e360–e367. doi:10.1016/S2542-5196(17)30156-0. PMC5729020 29276803

[r54] Luber G, McGeehin M. Climate change and extreme heat events. Am J Prev Med. 2008;35(5):429–435. 10.1016/j.amepre.2008.08.021 18929969

[r55] Weilnhammer V, Schmid J, Mittermeier I, et al. Extreme weather events in Europe and their health consequences: A systematic review. Int J Hyg Environ Health. 2021;233:113688. doi:10.1016/j.ijheh.2021.113688. 33530011

[r56] Hasan F, Marsia S, Patel K, Agrawal P, Razzak JA. Effective community-based interventions for the prevention and management of heat-related illnesses: A scoping review. Int J Environ Res Public Health. 2021;18(16):8362. doi:10.3390/ijerph18168362. PMC8394078 34444112

[r57] Gago EJ, Roldan J, Pacheco-Torres R, Ordóñez J. The city and urban heat islands: A review of strategies to mitigate adverse effects. Renew Sustain Energy Rev. 2013;25:749–758. doi:10.1016/j.rser.2013.05.057.

[r58] Sahani J, Kumar P, Debele SE. Efficacy assessment of green-blue nature-based solutions against environmental heat mitigation. Environ Int. 2023;179:108187. doi:10.1016/j.envint.2023.108187. Epub 2023 Sep 4. PMID: 37699297.

[r59] Stone B Jr, Vargo J, Liu P, Hu Y, Russell A. Climate change adaptation through urban heat management in Atlanta, Georgia. Environ Sci Technol. 2013;47(14):7780–7786. doi:10.1021/es304352e. Epub 2013 Jun 27. PMID: 23734623.

[r60] Song J, Pan R, Yi W, et al. Ambient high temperature exposure and global disease burden during 1990–2019: An analysis of the Global Burden of Disease Study 2019. Sci Total Environ. 2021;787:147540. doi:10.1016/j.scitotenv.2021.147540. Epub 2021 Jun 4. 33992940

[r61] Son JY, Lane KJ, Lee JT, Bell ML. Urban vegetation and heat-related mortality in Seoul, Korea. Environ Res. 2016;151:728–733. doi:10.1016/j.envres.2016.09.001. Epub 2016 Sep 17. PMID: 27644031; PMCID: . PMC5071166

[r62] Iungman T, Cirach M, Marando F, et al. Cooling cities through urban green infrastructure: A health impact assessment of European cities. Lancet. 2023;401(10376):577–589. doi:10.1016/S0140-6736(22)02585-5. Epub 2023 Jan 31. PMID: 36736334.

[r63] Venter ZS, Krog NH, Barton DN. Linking green infrastructure to urban heat and human health risk mitigation in Oslo, Norway. Sci Total Environ. 2020;709:136193. doi:10.1016/j.scitotenv.2019.136193. 31887497

[r64] Ballester J, Quijal-Zamorano M, Méndez Turrubiates RFet alHeat-related mortality in Europe during the summer of 2022. Nat Med. 2023;29(7):1857–1866. doi:10.1038/s41591-023-02419-z. Epub 2023 Jul 10. Erratum in: Nat Med. 2024;30(2):603. doi:10.1038/s41591-023-02649-1. PMID: 37429922; PMCID: . PMC10353926

[r65] Vicedo-Cabrera AM, Guo Y, Sera F, et al. Temperature-related mortality impacts under and beyond Paris Agreement climate change scenarios. Clim Change. 2018;150(3–4):391–402. doi:10.1007/s10584-018-2274-3. Epub 2018 Sep 13. PMID: 30405277; PMCID: . PMC6217994

[r66] Brochu P, Jimenez MP, James P, Kinney PL, Lane K. Benefits of increasing greenness on all-cause mortality in the largest metropolitan areas of the United States within the past two decades. Front Public Health. 2022;10:841936. doi:10.3389/fpubh.2022.841936. PMID: 35619828; PMCID: . PMC9127575

[r67] Nowak DJ, Hirabayashi S, Bodine A, Greenfield E. Tree and forest effects on air quality and human health in the United States. Environ Pollut. 2014;193:119–129. doi:10.1016/j.envpol.2014.05.028. Epub 2014 Jul 10. PMID: 25016465.

[r68] Anguelovski I, Connolly JJT, Cole H, et al. Green gentrification in European and North American cities. Nat Commun. 2022;13(1):3816. doi:10.1038/s41467-022-31572-1. PMID: 35780176; PMCID: . PMC9250502

[r69] Chen Y, Liu Z, Zhou BB. Population-environment dynamics across world's top 100 urban agglomerations: With implications for transitioning toward global urban sustainability. J Environ Manage. 2022;319:115630. doi:10.1016/j.jenvman.2022.115630. Epub 2022 Jul 11. PMID: 35834846.

[r70] Fastl C, Arnberger A, Gallistl V, Stein VK, Dorner TE. Heat vulnerability: Health impacts of heat on older people in urban and rural areas in Europe. Wien Klin Wochenschr. 2024;136(17–18):507–514. doi:10.1007/s00508-024-02419-0. Epub 2024 Aug 19. PMID: 39158652; PMCID: . PMC11390756

[r71] Fu Q, Zheng Z, Sarker MNI, Lv Y. Combating urban heat: Systematic review of urban resilience and adaptation strategies. Heliyon. 2024;10(17):e37001. doi:10.1016/j.heliyon.2024.e37001. PMID: 39281560; PMCID: . PMC11402234

[r72] Li Y, Svenning JC, Zhou W, et al. Global inequality in cooling from urban green spaces and its climate change adaptation potential. Preprint. Posted online July 2023.

[r73] Wu S, Chen B, Webster C, Xu B, Gong P. Improved human greenspace exposure equality during 21st century urbanization. Nat Commun. 2023;14(1):6460. doi:10.1038/s41467-023-41620-z. PMID: 37833296; PMCID: . PMC10575899

[r74] Chen B, Wu S, Song Y, Webster C, Xu B, Gong P. Contrasting inequality in human exposure to greenspace between cities of Global North and Global South. Nat Commun. 2022;13(1):4636. doi:10.1038/s41467-022-32258-4. PMID: 35941122; PMCID: . PMC9360024

[r75] Li Y, Svenning JC, Zhou W, et al. Green spaces provide substantial but unequal urban cooling globally. Nat Commun. 2024;15(1):7108. 10.1038/s41467-024-51355-0 PMC11369290 39223143

[r76] Dang TN, Van DQ Kusaka H, Seposo XT, Honda Y. Green space and deaths attributable to the urban heat island effect in Ho Chi Minh City. Am J Public Health. 2018;108(S2):S137–S143. doi:10.2105/AJPH.2017.304123, Epub 2017 Oct 26. PMID: 29072938; PMCID: PMC5922196

[r77] Norton BA, Coutts AM, Livesley SJ, Harris RJ, Hunter AM, Williams NS. Planning for cooler cities: A framework to prioritise green infrastructure to mitigate high temperatures in urban landscapes. Landscape Urban Plan. 2015;134:127–138. doi:10.1016/j.landurbplan.2014.10.018.

[r78] Farina G, Neverre N, Hérivaux C, Barriere J, Pinson S, Habarou H. How to account for spatial trade-offs in planning for urban climate adaptation? Optimizing green and grey infrastructures. J Environ Manage. 2024;372:123380. doi:10.1016/j.jenvman.2024.123380. Epub 2024 Nov 24. PMID: 39579571.

[r79] Stone B. Urban and rural temperature trends in proximity to large US cities: 1951–2000. Int J Climatol. 2007;27(13):1801–1807.

[r80] López-Bueno JA, Navas-Martín MA, Díaz J, et al. Analysis of vulnerability to heat in rural and urban areas in Spain: What factors explain heat's geographic behavior? Environ Res. 2022;207:112213. doi:10.1016/j.envres.2021.112213. Epub 2021 Oct 1. 34666017

