# Supplementary material for: The Role of Greenness in Heat-Related Mortality: A Systematic Review and Meta-Analysis
Source: Ann Glob Health. 2026 Sep 25;92(1):99. doi: 10.5334/aogh.5216 (PMC13614100; doi:10.5334/aogh.5216)
Supplement: Supplementary file 2 [file agh-92-1-5216-s2.pdf]

Supplementary Table S2

Table S2. List of the studies fully assessed for eligibility and excluded.

**Wrong exposition-analysis**	
**1.**	Zanobetti A, O'Neill MS, Gronlund CJ, Schwartz JD. Summer temperature variability and long-term survival among elderly people with chronic disease. Proc Natl Acad Sci U S A. 2012 Apr 24;109(17):6608-13.
**2.**	Xu Z, Tong S, Cheng J, Zhang Y, Wang N, Zhang Y, et al. Heatwaves, hospitalizations for Alzheimer's disease, and postdischarge deaths: A population-based cohort study. Environ Res. 2019;179:108782.
**3.**	Song J, Pan R, Yi W, Wei Q, Qin W, Song S, et al. Ambient high temperature exposure and global disease burden during 1990–2019: An analysis of the Global Burden of Disease Study 2019. Sci Total Environ. 2021;787:147540.
**4.**	Dang TN, Van DQ, Kusaka H, Seposo XT, Honda Y. Green space and deaths attributable to the urban heat island effect in Ho Chi Minh City. Am J Public Health. 2018;108(S2):S137–43.
**5.**	Burkart K, Meier F, Schneider A, Breitner S, Canário P, Alcoforado MJ, et al. Modification of heat-related mortality in an elderly urban population by vegetation (urban green) and proximity to water (urban blue): Evidence from Lisbon, Portugal. Environ Health Perspect. 2016;124(7):927–34
**6.**	Huang Z, Chan EY, Wong CS, Liu S, Zee BC. Health disparity resulting from the effect of built environment on temperature-related mortality in a subtropical urban setting. Int J Environ Res Public Health. 2022;19(14):8506.
**7.**	Schinasi LH, Bakhtsiyarava M, Sanchez BN, Kephart JL, Ju Y, Arunachalam S, et al. Greenness and excess deaths from heat in 323 Latin American cities: Do associations vary according to climate zone or green space configuration? Environ Int. 2023; 180:108230.
**8.**	Iungman T, Cirach M, Marando F, Pereira Barboza E, Khomenko S, Masselot P, et al. Cooling cities through urban green infrastructure: a health impact assessment of European cities. Lancet. 2023;401(10376):577–89
**9.**	López-Bueno JA, Díaz J, Sánchez-Guevara C, Sánchez-Martínez G, Franco M, Gullón P, et al. The impact of heat waves on daily mortality in districts in Madrid: The effect of sociodemographic factors. Environ Res. 2020; 190:109993.
**10.**	Madrigano J, Ito K, Johnson S, Kinney PL, Matte T. A case-only study of vulnerability to heat wave-related mortality in New York City (2000–2011). Environ Health Perspect. 2015;123(7):672–8.
**11.**	Song J, Lu Y, Zhao Q, Zhang Y, Yang X, Chen Q, et al. Effect modifications of green space and blue space on heat-mortality association in Hong Kong, 2008–2017. Sci Total Environ. 2022;838(Pt 2): 156127..
**12.**	Xu L, Ren C, Yuan C, Nichol JE, Goggins WB. An ecological study of the association between area-level green space and adult mortality in Hong Kong. Climate. 2017;5(55).
**13.**	Denpetkul T, Phosri A. Daily ambient temperature and mortality in Thailand: Estimated effects, attributable risks, and effect modifications by greenness. Sci Total Environ. 2021;791:148373.

**14.**	Kalkstein LS, Eisenman DP, de Guzman EB, Sailor DJ. Increasing trees and high-albedo surfaces decreases heat impacts and mortality in Los Angeles, CA. Int J Biometeorol. 2022;66(5):911–25..
**15.**	He Y, Cheng L, Bao J, Deng S, Liao W, Wang Q, et al. Geographical disparities in the impacts of heat on diabetes mortality and the protective role of greenness in Thailand: A nationwide case-crossover analysis. Sci Total Environ. 2020; 711:135098
**16.**	Song J, Gasparrini A, Wei D, Lu Y, Hu K, Fischer TB, et al. Do greenspaces really reduce heat health impacts? Evidence for different vegetation types and distance-based greenspace exposure. Environ Int. 2024; 191:108950.
**17.**	Hu K, Wang S, Fei F, Song J, Chen F, Zhao Q, et al. Modifying temperature-related cardiovascular mortality through green-blue space exposure. Environ Sci Ecotechnol. 2024; 20:100408.
**18.**	Taylor J, Simpson C, Brousse O, Viitanen AK, Heaviside C. The potential of urban trees to reduce heat-related mortality in London. Environ Res Lett. 2024;19(5):054004.
**19.**	Iungman T, Caballé SV, Segura-Barrero R, Cirach M, Mueller N, Daher C, et al. Co-benefits of nature-based solutions: A health impact assessment of the Barcelona Green Corridor (Eixos Verds) plan. Environ Int. 2025; 196:109313.
**20.**	Iungman T, Khomenko S, Barboza EP, Cirach M, Gonçalves K, Petrone P, et al. The impact of urban configuration types on urban heat islands, air pollution, CO2 emissions, and mortality in Europe: a data science approach. Lancet Planet Health. 2024;8(7):e489–e505.
**21.**	Du H, Yan M, Liu X, Zhong Y, Ban J, Lu K, et al. Exposure to concurrent heatwaves and ozone pollution and associations with mortality risk: A nationwide study in China. Environ Health Perspect. 2024;132(4): 47012.
**22.**	Wu Y, Wen B, Ye T, Huang W, Liu Y, Gasparrini A, et al. Estimating the urban heat-related mortality burden due to greenness: a global modelling study. Lancet Planet Health. 2025 Jul;9(7):101235. doi: 10.1016/S2542-5196(25)00062-2.
**23.**	Quick M, Duong M. Exploring the local impacts of heat on mortality: A small-area spatial analysis of Montreal, Toronto, and Vancouver, Canada. Can J Public Health. 2025. doi: 10.17269/s41997-025-01086-6.
**24.**	Cao Q, Yu Y, Sun S, Zhang Q, Liu C, Xia W, et al. Effect of built environment on the association between nighttime heat exposure and mortality in stroke patients: A case-crossover study in Shandong Province, China. *Ecotoxicol Environ Saf.*2025 Oct 1;304:119159. doi: 10.1016/j.ecoenv.2025.119159.
**Different exposure focus**	
**25.**	Goggins WB, Ren C, Ng E, Yang C, Chan EY. Effect modification of the association between meteorological variables and mortality by urban climatic conditions in the tropical city of Kaohsiung, Taiwan. Geospat Health. 2013;8(1):37–44.
**26.**	James P, Hart JE, Banay RF, Laden F. Exposure to greenness and mortality in a nationwide prospective cohort study of women. Environ Health Perspect. 2016;124(9):1344–52. doi:10.1289/ehp. 1510363..
**27.**	Orioli R, Antonucci C, Scortichini M, Cerza F, Marando F, Ancona C, et al. Exposure to residential greenness as a predictor of cause-specific mortality and stroke incidence in the Rome Longitudinal Study. Environ Health Perspect. 2019;127(2):027002. doi:10.1289/EHP2855.

**28.**	Santamouris M, et al. Heat mitigation technologies can improve sustainability in cities: An holistic experimental and numerical impact assessment of urban overheating and related heat mitigation strategies on energy consumption, indoor comfort, vulnerability and heat-related mortality and morbidity in cities. Energy Build. 2020; 217:110002.
**29.**	Chen H, et al. Residential greenness and cardiovascular disease incidence, readmission, and mortality. Environ Health Perspect. 2020;128(8):087005. doi:10.1289/EHP6161.
**30.**	Pereira Barboza E, Nieuwenhuijsen M, Ambròs A, Sá TH, Mueller N. The impact of urban environmental exposures on health: An assessment of the attributable mortality burden in Sao Paulo city, Brazil. Sci Total Environ. 2022; 831:154836. doi: 10.1016/j.scitotenv.2022.154836.
**31.**	Tsai WL, et al. Relationships between urban green land cover and human health at different spatial resolutions. Urban Ecosyst. 2019;22(2):315–24. doi:10.1007/s11252-018-0813-3.
**32.**	Jáuregui E, Tejeda A. Seasonal variations of the urban heat island at the surface and the near-surface and reductions due to urban vegetation in Mexico City. J Appl Meteorol Climatol. 2012;51(5):855–68.
**33.**	Zhang S, Breitner S, Stafoggia M, Donato FD, Samoli E, Zafeiratou S, et al. Effect modification of air pollution on the association between heat and mortality in five European countries. Environ Res. 2024;263(Pt 1):120023. doi: 10.1016/j.envres.2024.120023.
**34.**	Li M, Wen Y, Hu G. Relationship between green space and mortality in the cities of the Yangtze River Delta urban agglomeration. Forests. 2024;15(6):1066. doi:10.3390/f15061066.
**No mortality as outcome**	
**35.**	Bao J, Cao Z, Huang C, Lei L, Yang Y, Peng J, Shi X. Modification effect of urban landscape characteristics on the association between heat and stroke morbidity: A small-scale intra-urban study in Shenzhen, China. Sci Total Environ. 2021; 786:147466. doi: 10.1016/j.scitotenv.2021.147466.
**36.**	Guo X, Su W, Wang X, Hu W, Meng J, Ahmed MA, et al. Assessing the effects of air pollution and residential greenness on frailty in older adults: a prospective cohort study from China. Environ Sci Pollut Res Int. 2024;31(6):9091–105. doi:10.1007/s11356-023-31741-9. PMID: 38183550.
**37.**	Sha S, Cheng Q. Built or social environment? Effects of perceptions of neighbourhood green spaces on resilience of residents to heat waves. Urban For Urban Green. 2024; 94:128267. doi: 10.1016/j.ufug.2024.128267.
**38.**	Tuerdi N, Cao X, Tang H, Zhang Y, Zheng C, Wang X, et al. Combined effect of heatwaves and residential greenness on the risk of stroke among Chinese adults: A national cohort study. Ecotoxicol Environ Saf. 2025 Jul 1; 299: 118356. doi: 10.1016/j.ecoenv.2025.118356.
**39.**	Zhang J, Wang Y, Hu J, Zeng Q. Exposure to greenness modifies the association between extreme temperature events and ischemic stroke recurrence in Tianjin, China. *Int J Environ Health Res.* 2025. doi: 10.1080/09603123.2025.2502634.
**No direct % greenspace – mortality analysis**	

**40.**	Chaston TB, Broome RA, Cooper N, Duck G, Geromboux C, Guo Y, et al. Mortality burden of heatwaves in Sydney, Australia is exacerbated by the urban heat island and climate change: Can tree cover help mitigate the health impacts? Atmosphere. 2022;13(5):714. doi:10.3390/atmos13050714.
**41.**	Tan J, Zheng Y, Song G, Kalkstein LS, Kalkstein AJ, Tang X. Heat wave impacts on mortality in Shanghai, 1998 and 2003. Int J Biometeorol. 2007;51(3):193–200. doi:10.1007/s00484-006-0070-y.
**42.**	Kim EJ, Kim H. Effect modification of individual- and regional-scale characteristics on heat wave-related mortality rates between 2009 and 2012 in Seoul, South Korea. Sci Total Environ. 2017;595:141–8. doi:10.1016/j.scitotenv.2017.03.248.
