# Supplementary material for: The Role of Greenness in Heat-Related Mortality: A Systematic Review and Meta-Analysis
Source: Ann Glob Health. 2026 Sep 25;92(1):99. doi: 10.5334/aogh.5216 (PMC13614100; doi:10.5334/aogh.5216)
Supplement: Supplementary file 3 [file agh-92-1-5216-s3.pdf]

Supplementary Table S3. Methodological quality of the included studies *(Yes=Green, No=Red, Not Reported=Yellow, Not Applicable=Blue).*

	Choi et al. 2022	Choi et al. 2024	Gronlund et al. 2015	Hu et al. 2025	L. Luque García et	Pascal et al. 2021	Qiu et al. 2021	Song et al. 2023	Wicki et al. 2024	Zhang et al. 2021	Zhang et al. 2024
1. Clearly stated research question	Green	Green	Green	Green	Green	Green	Green	Green	Green	Green	Green
2. Clearly specified and defined study population	Green	Green	Green	Green	Green	Green	Green	Green	Green	Green	Green
3. Participation rate of eligible persons at least 50%	Yellow	Green	Yellow	Blue	Red	Green	Red	Green	Green	Yellow	Yellow
4. All subjects selected from similar populations	Green	Green	Green	Green	Green	Green	Green	Green	Green	Green	Green
5. Justification of sample size or statistical power provided.	Red	Blue	Yellow	Yellow	Red	Green	Red	Green	Yellow	Yellow	Green
6. Exposures measured prior to measuring outcomes	Green	Green	Green	Green	Green	Green	Green	Green	Green	Green	Green
7. Adequate time period to expect an association between exposure and outcome.	Green	Green	Green	Green	Green	Green	Green	Green	Green	Green	Green
8. Examination of different levels of exposure	Green	Green	Green	Green	Green	Green	Green	Green	Red	Green	Green
9. Clearly defined, valid and reliable measures of exposure.	Green	Green	Green	Green	Green	Green	Green	Green	Green	Green	Green
10. Assessment of exposures more than once over time.	Yellow	Red	Yellow	Green	Yellow	Green	Red	Green	Red	Green	Yellow
11. Clearly defined, valid and reliable outcome measures.	Green	Green	Green	Green	Green	Green	Green	Green	Green	Green	Green
12. Outcome assessors blinded to exposure status	Yellow	Yellow	Yellow	Blue	Yellow	Green	Blue	Yellow	Yellow	Yellow	Yellow
13. Loss to follow-up after baseline ≤ 20%	Blue	Blue	Yellow	Blue	Green	Green	Yellow	Green	Blue	Green	Blue
14. Statistical adjustment for key confounding variables	Green	Green	Green	Green	Green	Green	Green	Green	Green	Green	Green
