# Supplementary material for: The Role of Greenness in Heat-Related Mortality: A Systematic Review and Meta-Analysis
Source: Ann Glob Health. 2026 Sep 25;92(1):99. doi: 10.5334/aogh.5216 (PMC13614100; doi:10.5334/aogh.5216)
Supplement: Supplementary file 4 [file agh-92-1-5216-s4.pdf]

Supplementary Table S4

Table S4. Meta-bias for the associations between greenness and mortality during extreme heat periods.

Level of greenness	n	Intercept	Estimate (SE)	P value
**Low Greenness**	11	5.2008	5.2008 (5.9893)	0.408
**High Greenness**	11	0.6317	0.6317 (3.0217)	0.839

n represents the number of studies included in each analysis.
Abbreviations: SE, standard error.
