# Supplementary material for: The Role of Greenness in Heat-Related Mortality: A Systematic Review and Meta-Analysis
Source: Ann Glob Health. 2026 Sep 25;92(1):99. doi: 10.5334/aogh.5216 (PMC13614100; doi:10.5334/aogh.5216)
Supplement: Supplementary file 5 [file agh-92-1-5216-s5.pdf]

Supplementary Figure S1

Figure S1. Sensitivity Analysis of the Association Between Low Greenness Exposure and mortality during extreme heat periods.

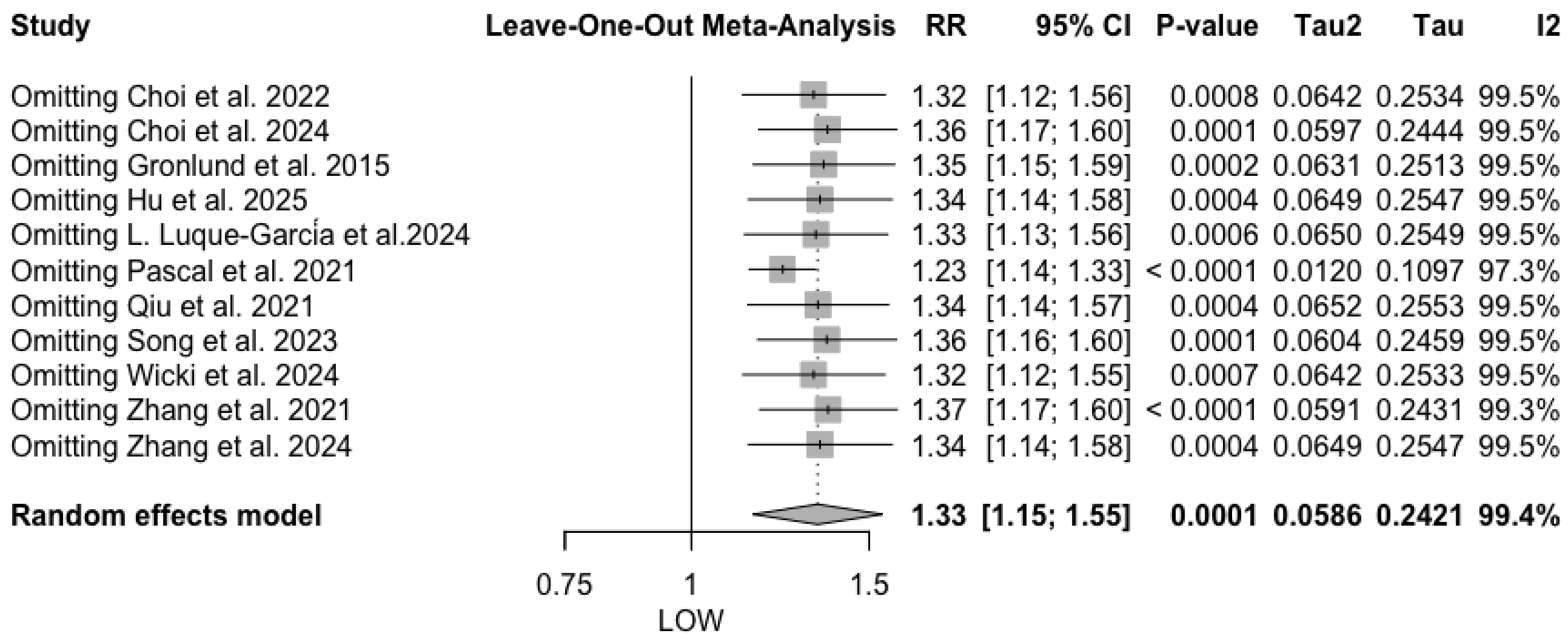

Abbreviations: CI, confidence interval.
I^2 represents heterogeneity.
