# Supplementary material for: The Role of Greenness in Heat-Related Mortality: A Systematic Review and Meta-Analysis
Source: Ann Glob Health. 2026 Sep 25;92(1):99. doi: 10.5334/aogh.5216 (PMC13614100; doi:10.5334/aogh.5216)
Supplement: Supplementary file 6 [file agh-92-1-5216-s6.pdf]

Supplementary Figure S2

Figure S2. Sensitivity analysis of the association between high greenness exposure and mortality during extreme heat periods.

Study	Leave-One-Out Meta-Analysis	RR	95% CI	P-value	Tau2	Tau	I2
Omitting Choi et al. 2022		1.18	[1.01; 1.37]	0.0376	0.0577	0.2401	98.1%
Omitting Choi et al. 2024		1.20	[1.04; 1.40]	0.0144	0.0530	0.2302	98.0%
Omitting Gronlund et al. 2015		1.20	[1.04; 1.40]	0.0152	0.0534	0.2311	98.0%
Omitting Hu et al. 2025		1.18	[1.01; 1.37]	0.0377	0.0577	0.2401	98.0%
Omitting L. Luque-García et al.2024		1.17	[1.00; 1.36]	0.0447	0.0569	0.2385	98.1%
Omitting Pascal et al. 2021		1.11	[1.03; 1.19]	0.0050	0.0111	0.1056	97.1%
Omitting Qiu et al. 2021		1.19	[1.03; 1.38]	0.0177	0.0543	0.2331	98.1%
Omitting Song et al. 2023		1.20	[1.04; 1.40]	0.0155	0.0536	0.2314	98.0%
Omitting Wicki et al. 2024		1.17	[1.00; 1.36]	0.0450	0.0566	0.2379	98.1%
Omitting Zhang et al. 2021		1.20	[1.03; 1.39]	0.0213	0.0556	0.2359	95.6%
Omitting Zhang et al. 2024		1.18	[1.01; 1.37]	0.0397	0.0576	0.2400	97.5%
**Random effects model**		**1.18**	**[1.03; 1.36]**	**0.0202**	**0.0516**	**0.2272**	**97.9%**

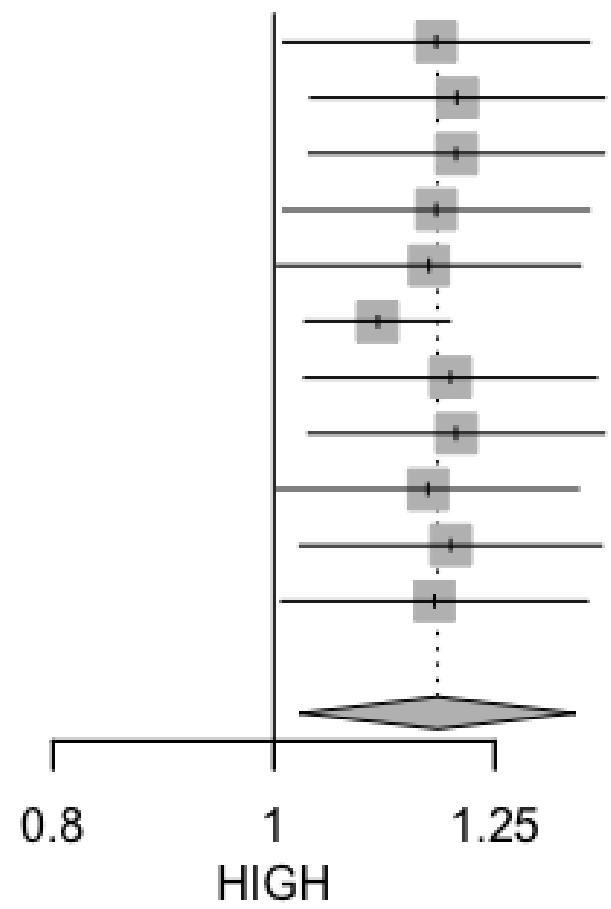

Abbreviations: CI, confidence interval.
I^2 represents heterogeneity.
