# Supplementary material for: The Role of Greenness in Heat-Related Mortality: A Systematic Review and Meta-Analysis
Source: Ann Glob Health. 2026 Sep 25;92(1):99. doi: 10.5334/aogh.5216 (PMC13614100; doi:10.5334/aogh.5216)
Supplement: Supplementary file 7 [file agh-92-1-5216-s7.pdf]

Supplementary Figure S3

A)

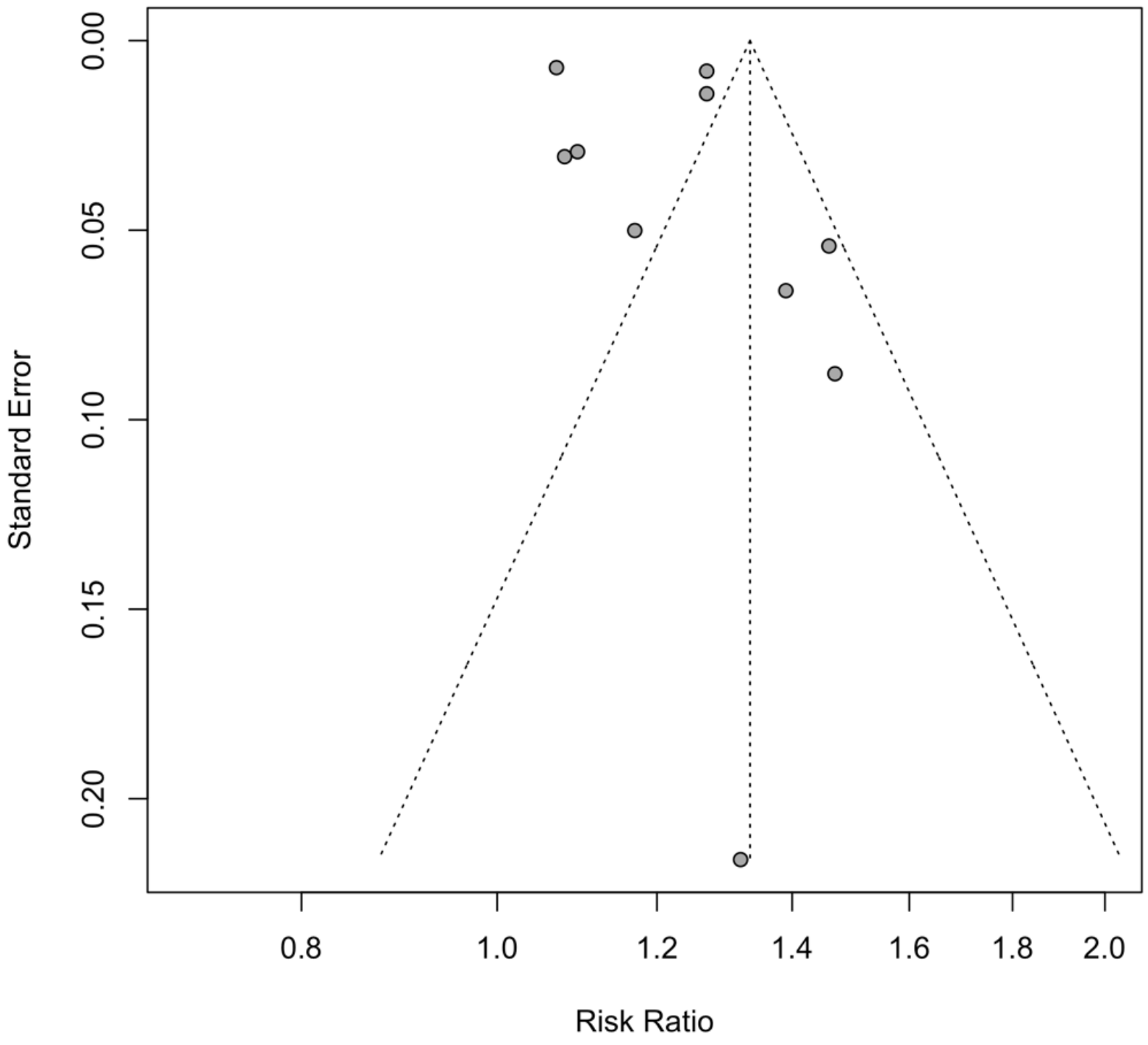

B)

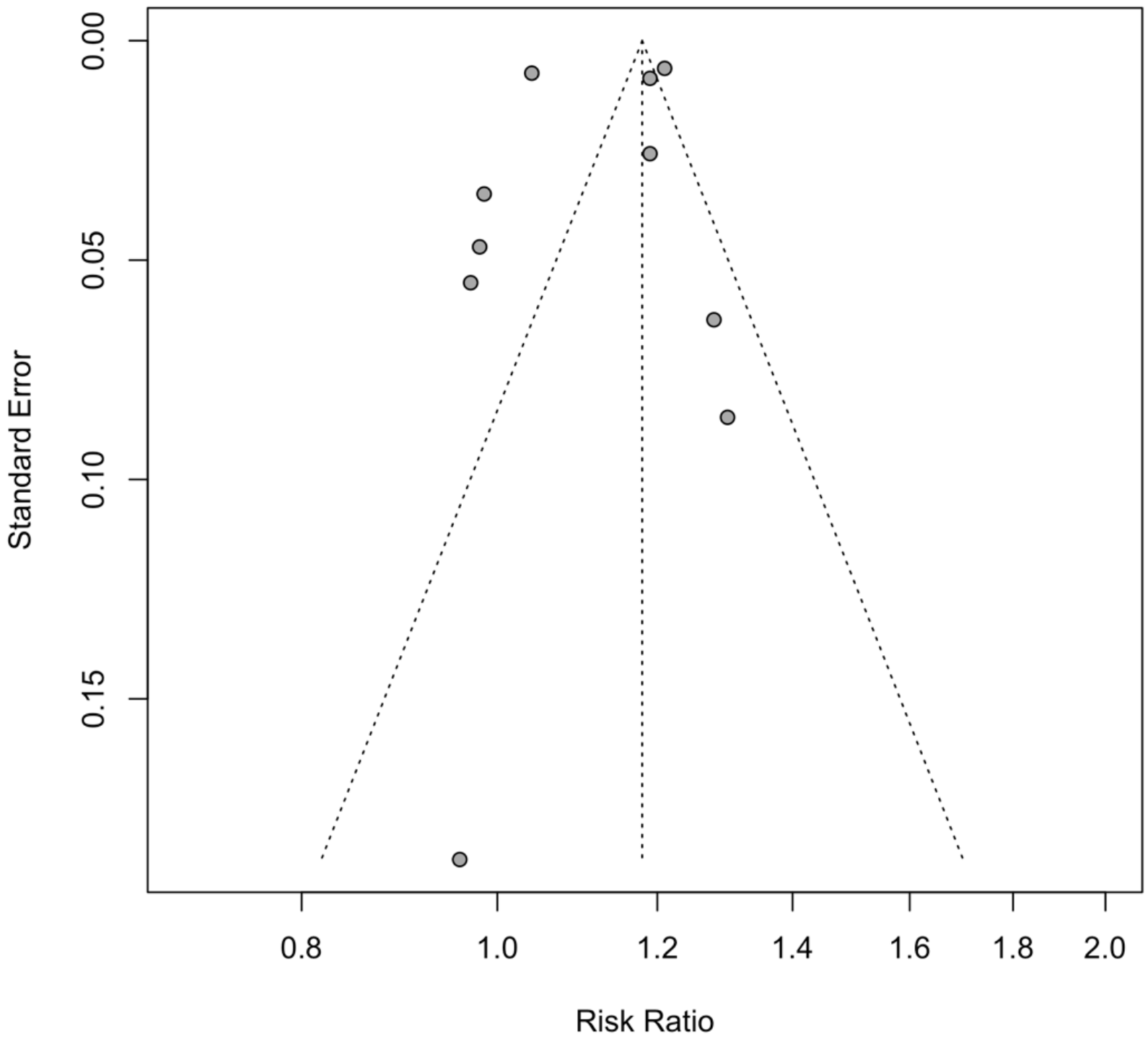

Figure S3. Publication bias for the associations of low (A) and high (B) greenness with mortality risk during extreme heat periods.
